# Primary Progressive Multiple Sclerosis: Putting Together the Puzzle

**DOI:** 10.3389/fneur.2017.00234

**Published:** 2017-05-31

**Authors:** Ahmed Abdelhak, Martin S. Weber, Hayrettin Tumani

**Affiliations:** ^1^Department of Neurology, Ulm University, Ulm, Germany; ^2^Department of Neuropathology, University Medical Center, Georg August University, Göttingen, Germany; ^3^Department of Neurology, University Medical Center, Georg August University, Göttingen, Germany; ^4^Specialty Clinic of Neurology Dietenbronn, Schwendi, Germany

**Keywords:** primary progressive multiple sclerosis, pathophysiology, treatment, Epstein–Barr virus, risk factors

## Abstract

The focus of multiple sclerosis research has recently turned to the relatively rare and clearly more challenging condition of primary progressive multiple sclerosis (PPMS). Many risk factors such as genetic susceptibility, age, and Epstein–Barr virus (EBV) infection may interdepend on various levels, causing a complex pathophysiological cascade. Variable pathological mechanisms drive disease progression, including inflammation-associated axonal loss, continuous activation of central nervous system resident cells, such as astrocytes and microglia as well as mitochondrial dysfunction and iron accumulation. Histological studies revealed diffuse infiltration of the gray and white matter as well as of the meninges with inflammatory cells such as B-, T-, natural killer, and plasma cells. While numerous anti-inflammatory agents effective in relapsing remitting multiple sclerosis basically failed in treatment of PPMS, the B-cell-depleting monoclonal antibody ocrelizumab recently broke the dogma that PPMS cannot be treated by an anti-inflammatory approach by demonstrating efficacy in a phase 3 PPMS trial. Other treatments aiming at enhancing remyelination (MD1003) as well as EBV-directed treatment strategies may be promising agents on the horizon. In this article, we aim to summarize new advances in the understanding of risk factors, pathophysiology, and treatment of PPMS. Moreover, we introduce a novel concept to understand the nature of the disease and possible treatment strategies in the near future.

## Introduction

Since first described by Charcot almost two centuries ago (1), the field of multiple sclerosis (MS) witnessed enormous advancements. Not only we know better about the pathophysiology of the disease but also the different risk factors, clinical subtypes, and at last but not at least how attenuate the pathophysiological processes leading to neuronal demise. The success cannot be better seen than in the field of relapsing remitting multiple sclerosis (RRMS) with more than six medications got approved by the FDA and EMA since 2010 (2). However, the primary progressive multiple sclerosis (PPMS) remains a considerable challenge. Despite having more than one study fulfilling its primary end point (3, 4) to the time of writing this article, we still do not have any approved medication.

Nevertheless, the recent focus on progressive MS forms [primary and secondary progressive multiple sclerosis (PP and SPMS)] leads to a deep insight in the different pathological aspects driving the disease. In this article, we summarize the recent developments and integrate the different aspects to provide a framework explaining the sequence of events leading to the unique clinical picture.

## Pathophysiology of MS

### Genetic Factors

The genetic predisposition for MS is based on observations highlighting the role of maternal genes and epigenetic factors (5). Half siblings from the same mother and first degree relatives have a higher MS incidence (6). HLA-DR2 haplotype demonstrated association and linkage to MS (7). HLA-DRB1*15:01 allele is associated with lower age of onset, white matter lesions (WMLs) volume, and reduction of parenchymal volume in RRMS (8). Lack of difference in HLA-status between RRMS and PPMS patients suggests that DRB1-related mechanisms are contributing to both phenotypes (7). Furthermore, many loci variants outside MHC have been correlated to MS risk and involve pathways of like kappa-light-chain-enhancer’s of activated B-cells (NF-KappaB) mediated cytokine release and activation of immune cells both in the periphery and inside the central nervous system (CNS) (9, 10). Different loci variants may correlate with relapse rate (11) and cervical cord atrophy (12) indicating a possible role in evolving of the disease phenotype. Nevertheless, no loci so far have been associated with a specific clinical subtype like PPMS.

### Environmental Factors

The most important environmental factor is the sun exposure and its subsequent effect on vitamin D deficiency (13). The role of vitamin D is prominent only in RRMS, where serum levels of vitamin D are low and correlate with relapse rates (14). PPMS patients have normal levels of vitamin D (15) with no correlation with disability progression (16). Vitamin D interacts with HLA-DRB1*1501 (17) influencing the proliferation, maturation and function of different immune cells (15). The abovementioned findings make it tempting to postulate a role of vitamin D in the evolution of the clinical phenotype of MS.

### Epstein–Barr Virus (EBV) Infection

Epstein–Barr virus is involved in different pathophysiological aspects of MS; RRMS patients are more frequently EBV seropositive than controls, and the risk of MS increases in seronegative individuals dramatically after the seroconversion (18). Delayed primary EBV infection and MS share many epidemiological features like socioeconomic status, latitudinal variation, and effect of migration (19). Elevated anti-Epstein–Barr nuclear antigen 1 IgG was found also in the PPMS patients and was associated with MRI disease activity (20). EBV infects B cells leading to their maturation into latently infected, apoptosis-resistant memory B cells (21). EBV-infected B cells are found in the meningeal infiltration, perivascularly, WMLs and in cervical lymph nodes in PPMS (22). EBV involvement in the disease is extensively reviewed by Pender and Burrows (23). EBV induces autoreactive B cells formation (B_Auto_) through cross reactivity with some myelin, bystander damage during the immune reaction against EBV infection, immune reaction against αβ crystallin expressed by oligodendrocytes, and at last infection of some naturally present autoreactive B cells leading to their maturation and initiating the immune cascade in CNS (23).

The immune reaction to EBV infection in healthy older subjects (>50) is characterized by secretion of IFN-γ and IL-6 leading to a chronic inflammatory state with progressive activation of tissue-resident macrophages and monocytes (24), an immune state similar to PPMS. However, these similarities should be confirmed in further studies.

### Age

Progressive phase of MS (PP and SPMS) is the result of long-lasting degenerative changes, which appears only when an age threshold is reached and progresses in similar rates (25). Generally, older MS patients exhibit less focal inflammation (26), with more frequent motor, brainstem, and cerebellar manifestations associated with limited recovery capacities in RRMS (27) making the distinction between incompletely resolving acute attacks and progressive worsening according to the current definition of relapses very challenging.

### Gut Microbiome

Gut flora can provoke autoreactive CD4+ formation through antigenic mimicry, mostly with myelin oligodendrocytes glycoprotein or through innate immune signaling (28). Germ-free EAE mice were protected from the development of inflammatory lesions in brain with marked reduction in Th17 cells. Recolonization resulted in restoration of the Th17 and development of the EAE symptoms (29). A concrete role of the gut microbiome in PPMS is still unknown.

## Pathological Changes

The predominant lesions in PPMS are slowly expanding lesions with T cells, microglial, and macrophage-associated demyelination in close similar to pattern 1 demyelination (30). While the involvement of different CD4+ subtypes (Th1, Th17, and Th9) is one of the very initial events in MS (31), the main lymphocytes to be found in the lesions are CD8+ cells and correlate with the degree of axonal damage (32). sCD27, a marker of intrathecal inflammation secreted mainly by T cells, is elevated in PPMS (33). Prominent T_FH_ and Th17 activation in serum of PPMS patients was reported and correlated with the progression rate (34).

Evidences for B cell involvement in PPMS are numerous: the intrathecal IgG production, the detection of B cells within MS lesions, meningeal infiltrate, perivascular space and MS parenchyma, the presence of autoreactive antibodies against myelin and its products (32), and finally the success of B cell-based therapies in PPMS (35). B cells are scattered in the meninges in a diffuse manner with tertiary lymphoid follicles formation only in aggressive disease with active progressive disease (36). B- and plasma cells in PPMS lesions correlate with the severity of axonal damage (26). B cells are pathogenic through multiple pathways including antigen presentation, cytokines release, and producing of the autoantibodies (37). Their role beyond the synthesis of autoantibodies is confirmed by the fact that highly effective monoclonal anti CD-20 antibodies do not eliminate the long-lasting antibodies producing plasma cells (37). One example for non-antigen-presenting B cells is the pro-inflammatory granulocyte macrophage colony-stimulating factor (GM-CSF) B cells; through their GM-CSF secretion they induce pro-inflammatory myeloid cell response promoting the release of Th1- and Th17-differentiating cytokines like IL-6 and -12 (38).

The discovery of the B_reg_ cells secreting IL-10, IL-35, and TGF-b indicates the complex role of B cells in MS. B_reg_ can restore Th1/Th2 balance, inhibit Th1 and Th17 cell differentiation, and inhibit macrophages (37). Moreover, the secreted antibodies may play a role in regulating the immune system and inducing remyelination (39).

Phagocytic cells like the macrophages are the most common cells found in the slowly expanding lesions in PPMS (30). They are derived from blood monocytes and migrate into CNS after stimulation in the blood (40). The pro-inflammatory M1 play central role both in the demyelination and axonal damage through reactive oxygen spices, nitric oxide, and glutamate (40). CNS-infiltrating macrophages were able to induce progressive EAE through sustained secretion of TNF (41). Levels of sCD14, a marker of macrophageal activity, were higher in patients with PPMS compared to healthy controls, but similar to RRMS patients in relapses but not in remission (42). Nevertheless, macrophages (anti-inflammatory M2) are essential for the remyelination by clearing the damaged tissues in the lesions (40).

Dendritic cells can also be found in MS lesions (32). Dendritic cells form SPMS patients secret much higher levels of IL-18 than those from RRMS patients and healthy controls (43) and induce—*in vitro*—solely a Th1 cell response not Th1 and Th2 like in RRMS suggesting a role of dendritic cells in the disease transition into the progressive phase (44). Based on the similarities between SP and PPMS, a role of dendritic cells in PPMS cannot be excluded.

Over the last years, microglial activation (MiA) gained more interest as one of the key mechanisms for neurodegeneration and axonal demise in MS (30). In PPMS, the microglia were diffusely active in the lesions and in normal-appearing white matter (NAWM) and normal-appearing gray matter (NAGM) (45). Activated microglia in NAWM forms microglial nodules in close proximity to stressed oligodendrocytes and degenerated axons with profound release of oxygen-free radicals (30). MiA in cortical gray matter of SPMS is caused by the diffusion of inflammatory mediators from the meninges, especially from meningeal B cell infiltration and strongly correlates with clinical disability scores (46).

Similarly, the astrocytes are considered of particular importance in MS. Besides there well-known role in “scar formation,” recently the astrocytes have been identified as a potent secretor of different pro-inflammatory cytokines making them a possible target for therapeutic interventions (30).

### White Matter Damage

Radiologically the white matter pathology in PPMS is divided into three categories as follows.

#### White Matter Lesions

The well-defined hyperintense T2 WMLs indicate local demyelination of the WM. Histopathologically, the WMLs are either active with hypercellular infiltrate, chronic active or inactive. Both active and chronic active lesions are characterized by relative preservation of the axons but the cellular infiltrate differs; in the former, the lymphocytes are the main cells whereas in the latter the myelin-laden microphages form the mainstay of the lesions. On the other hand, the inactive lesions are characterized by extensive astrogliosis (47).

#### Diffusely Abnormal White Matter

Diffusely abnormal white matter (DAWM) refers to the diffuse and subtle signal hyperintensities in the WM. The DAWM in PMS exhibits no acute changes like demyelination or blood–brain barrier (BBB) leakage, nonetheless chronic axonal degeneration and gliosis. DAWM most likely represents degenerative changes secondary to remote focal WM pathologies (48).

#### Normal-Appearing White Matter

Normal-appearing white matter exhibits normal signal in the conventional T2 sequences. The NAWM changes include axonal injury without demyelination, low-grade inflammation, microglial, and astrocytic activation without being correlated to the WML load excluding the possibility that they are “pure” secondary retrograde axonal degeneration. The degree of axonal loss in NAWM as well as white matter atrophy measurements correlate with disease severity in SPMS (49).

### Gray Matter Damage (GMD)

Gray matter damage emerged over the last years as a major determinant of disability and disease progression (50). GMD involves different lesion types with damage of NAGM (51). Possible mechanisms are retrospective degenerative changes, inflammatory infiltrate in the meninges, MiA, iron accumulation, and primary oligodendrocytic degeneration (51). GM atrophy correlate better with the long-term disability than WMLs (52).

## The Role of Mitochondrial Dysfunction in PPMS

Mitochondrial dysfunction and energy deficits gained interest as a main mechanism of neuronal demise (53). The mitochondrial dysfunction with subsequent cellular hypoxia is especially relevant for the neurodegeneration of susceptible chronically demyelinated axons commonly found in PMS through energy failure, induction of apoptosis, and enhanced production of oxygen species (53). Corresponding to that, positive correlation between CSF lactate and disease progression was reported in RRMS patients (54). We confirmed a similar correlation in PPMS patients in a large multicentric CSF cohort including 254 PPMS patients (unpublished data). A positive correlation between CSF lactate and number of inflammatory MS plaques was reported in another study with 33 clinically isolated syndrome (CIS) patients (55). Another marker, the level *N*-acetylaspartate (NAA) measured using MRI spectroscopy, was reduced in MS patients and correlated to clinical severity of the disease (56).

## Role of Iron

Iron accumulation in PMS is an age-dependent process leading to free-radicals’ release, glutamate toxicity, and exacerbation of the neuronal demise (57). Iron-induced T2/hypointensities were reported in the GM, WML, and periventricularly around the veins (58) and is correlated with disease progression even better than brain atrophy (59). Iron deposition in deep gray matter was elevated in SPMS patients compared to controls (60). Furthermore, the iron-storage protein “ferritin” and soluble transferrin receptors were elevated in the CSF and serum of SPMS patients compared to controls (61). A similar role can be postulated in PPMS.

## The Mechanism of Axonal Degeneration and the Relationship Between The Neuroinflammation and Neurodegeneration in PPMS

Over the last years, two main hypotheses were postulated to explain the neuronal demise in MS (62): the inflammation-induced neurodegeneration and the neurodegeneration-provoked inflammation.

The inflammation-induced demyelination leads to death loss and subsequent neurodegeneration as in EAE models (63). APP axonal spheroids indicating transected injured axons are correlated with T and B cell infiltrates in MS lesions (26). Furthermore, early focal inflammatory lesions are associated with higher density of transected axons than in the later phases of the disease (64). Moreover, the meningeal inflammation is correlated with the cortical axonal loss and seems to be the driving force for active demyelination as well as neuronal, axonal, and synaptic destruction in the cerebral cortex of MS patients (65).

The second hypothesis postulates an autonomous degeneration of oligodendrocytes and myelin followed by MiA and subsequently invasion of inflammatory cells (66).

A combination of both mechanisms where the low-degree inflammation provides constant insult to the susceptible oligodendrocytes or dysfunctional axon–glial unit like in cases of disturbed iron metabolism, glutamate homeostasis, and mitochondrial dysfunction cannot be excluded (67). Nevertheless, the success of the anti CD-20 in slowing the disease progression emphasizes the role of the inflammation leading to emergence of the first hypothesis as a valid explanation for the neuronal demise in PPMS (68).

## Summary of the Sequence of Pathological Events in MS

**Figure 1:**
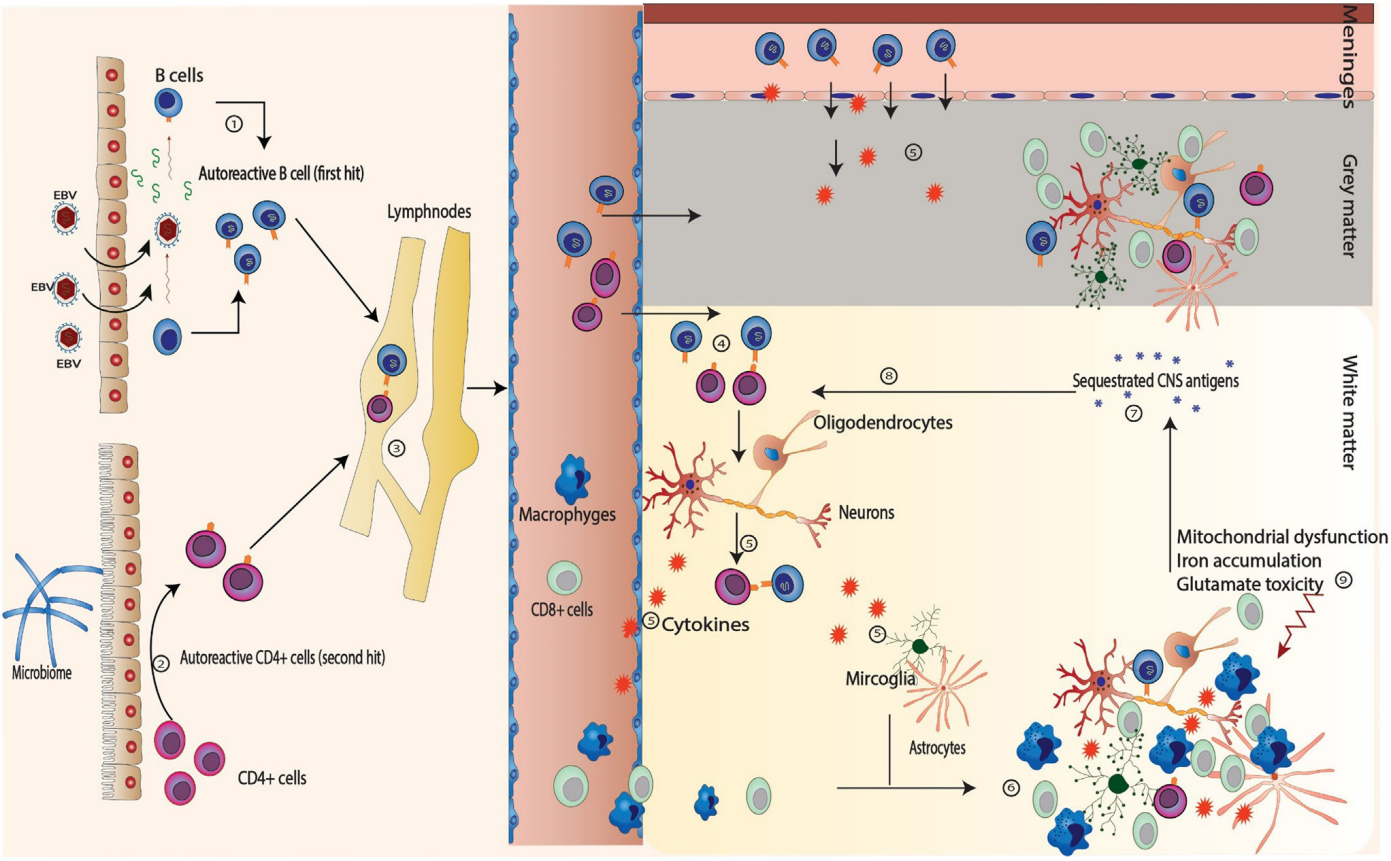
**Sequence of events in multiple sclerosis**. (1) Autoreactive B (B_Auto_) cells are formed by epitopes mimicry with Epstein–Barr virus (EBV) antigens or through persistent activation of the naturally presence autoreactive B cells through the chronic EBV infection (*first hit*). (2) Autoreactive CD4+ T (T_Auto_) cells are formed through antigen mimicry with intestinal flora (*second hit*). (3) The autoreactive B and CD4+ T cells interact in the peripheral lymph nodes leading further activation. (4) After releasing into blood stream, they both cross the blood–brain barrier and interact again in the perivascular space. (5) They recognize the self-antigens sequestrated in the central nervous system and release cytokines to attract other inflammatory cells (macrophages, cytotoxic CD8+) from the blood as well as to stimulate the microglia and astrocytes.(6) and (7) The inflammatory cells attack the neurons and the oligodendrocytes leading to demyelination, neuronal death with release of many sequestrated intracellular antigens (*first event*). (8) These antigens provoke more B and T cells reaction leading to accentuation of the inflammatory cascade (*second event*). (9) Other factors like mitochondrial dysfunction, glutamate cytotoxicity, and iron accumulation play import role in the demise of neurons, especially in primary progressive multiple sclerosis and SPMS.

**Figure 2:**
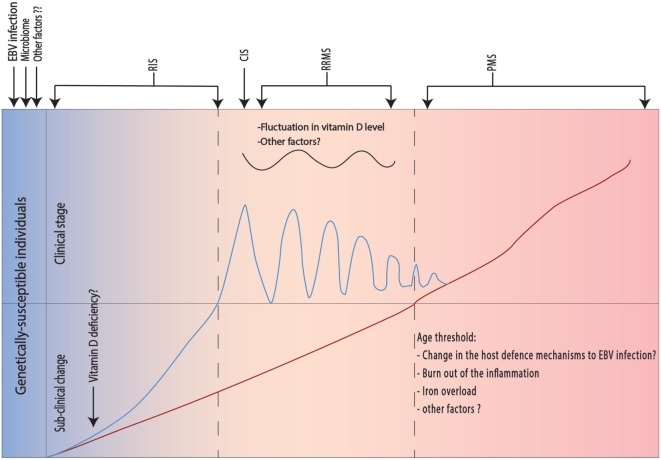
**Different risk factors and their effect on the clinical phenotype in multiple sclerosis (MS)**. The pathological process in MS occurs in genetically susceptible individuals after in presence of the Epstein–Barr virus (EBV) infection and predisposing intestinal microbiome (see below). Initially, the pathological process does not lead to clinical manifestations, but radiological changes may be present [radiologically isolated syndrome (RIS)]. The presence of vitamin D deficiency will exacerbate the inflammatory changes leading to appearance of the first relapse [clinically isolated syndrome (CIS)], which is usually followed by complete recovery. Fluctuation in vitamin D levels and eventually other unknown factors will lead to appearance of further relapses and starting of the relapsing remitting multiple sclerosis (RRMS). Another slowly inflammatory triggered neurodegenerative process takes place in the background and exhibits its clinical manifestation only after exceeding an age threshold. One possible explanation for the age threshold is known changes in the host defense to EBV infection. Other factors like age-dependent local iron precipitation may play a role.

Multiple sclerosis is an autoimmune disease involving both autoreactive B and T cells. EBV infection induces formation of autoreactive B cells (B_Auto_) either through antigen mimicry or through infection of the normally present B_Auto_-forming apoptosis-resistant active memory B_Auto_ cells (*first hit*). On the other hand, autoreactive CD4+ T cells (T_Auto_) are induced by the intestinal microbiome (*second hit*). Both B_Auto_ and T_Auto_ interact in the peripheral lymphoid tissue. T_Auto_ cells cross the BBB and are further activated by perivascular B_Auto_ sells. B and T cells recognize the neuronal antigens and start an inflammatory reaction leading to migration of CD8+ cells and macrophages through BBB as well as activation of the microglial cells and astrocytes leading to demise of the neurons (*first event*). The apoptotic neurons and oligodendrocytes release other sequestrated antigens that subsequently will be recognized by B and T cells accentuating the inflammatory reaction (*second event*) with wide spread low-grade inflammatory process. Other factors like mitochondrial dysfunction, glutamate cytotoxicity, and iron accumulation lead to further demise of neurons (Figure 1). We postulate that different predisposing factors may not only affect the MS course directly but also interact with each other leading to further complexification of the pathophysiology of the disease and might contribute to the determination of the clinical phenotype of the disease by focal accentuation of the inflammatory reaction with the clinical symptoms of an exacerbation (Figure 2).

## Current and Possible Future Treatments

Most clinical trials in PPMS were disappointing: from methylprednisolone (69), through glatiramer acetate (70), rituximab (71), interferon-beta (72, 73), and at last fingolimod (74). Lack of efficacy, inappropriate patients’ selection, short study period, and non-optimal primary outcome are the major causes of the negative results (75). The ORATORIO study was the first phase 3 study to meet its primary endpoint in PPMS (4) (Table 1). In the following section, we try to summarize the most promising treatments in PPMS.

**Table 1:** **Overview of the major clinical trials in primary progressive multiple sclerosis**.

Substance (mode of action)	Study name	Study design	Patients	Study duration	Results regarding disease progression
Interferon b-1a (immunomodulatory)	–	SC, R, DB, Plc-Ctrl	50	2 years	There was no significant difference in disease progression between the individual or combined treatment arms and placebo (72)

Glatiramer acetate (immunomodulatory)	PROMiSe	Phase 3, MC, R, DB, Plc-Ctrl	943	3 years	The tendency for delay in the time to sustained progression of accumulated disability in GA-treated patients compared with PBO-treated patients did not achieve statistical significance {hazard ratio, 0.87 [95% confidence interval (CI), 0.71–1.07]; *p* = 0.1753} (70)

Interferon b-1b (immunomodulatory)	–	Phase 2, SC, R, DB, Plc-Ctrl	73	2 years	Time to neurological deterioration confirmed on two consecutive visits (3 months) was not different between trial arms (treatment arm 65.8% versus placebo arm 56.8%; *p* = 0.3135) (73)

Rituximab (anti-CD20, B-cell depletion)	OLYMPUS	Phase 2/3, MC, R, DB, Plc-Ctrl	439	2 years	There was no evidence of significant difference in time to CDP between the rituximab and placebo groups (*p* = 0.1442) (71)

Fingolimod (immunomodulatory)	INFORMS	Phase 3, MC, R, DB, Plc-Ctrl	970	Up to 5 years	Fingolimod showed no difference compared with placebo (hazard ratio 0.95, 95% CI 0.80–1.12; *p* = 0.544) in the time to 3-month CDP (74)

MD1003 (biotin) (remyelinating agent)	MS-SPI	Phase 2/3, MC, R, DB, Plc-Ctrl	154	1 year	A total of 13 (12.6%; 95% CI: 6.9–20.6%) patients treated with MD1003 had a reduction in multiple sclerosis-related disability at month 9, confirmed at month 12, compared with none in the placebo arm (3)

Ocrelizumab (anti-CD20, B-cell depletion)	ORATORIO	Phase 3, MC, R, DB, Plc-Ctrl	732	3 years	The percentage of patients with 12-week confirmed disability progression (primary end point) was 32.9% with ocrelizumab versus 39.3% with placebo (hazard ratio, 0.76; 95% CI, 0.59–0.98; relative risk reduction, 24%; *p* = 0.03) (4)

Laquinimod (immunomodulatory, neuroprotective)	ARPEGGIO	Phase 2, MC, R, DB, Plc-Ctrl	374	Ongoing	Ongoing (76)

*SC, single center; MC, multicenter; R, randomized; DB, double-blinded; Plc-Ctrl, placebo-controlled*.

### Ocrelizumab

The immunomodulatory agent “Ocrelizumab” is a humanized monoclonal anti-CD20 antibody. It attacks different epitopes on pre-B cells and memory B rendering it better tolerable and possibly more effective than Rituximab (77). Ocrelizumab acts mainly against antigen-presenting and cytokine-releasing B cells not stem cells or plasma cells (68). The resulting B cell depletion is mediated either through compliments, cytotoxic CD8+ or induced apoptosis (78).

Ocrelizumab is the first drug ever to show efficacy in slowing the disease progression in a phase 3 clinical trial with PPMS patients (79). In the double-blinded, placebo-controlled study “ORATORIO” with 732 PPMS patients, ocrelizumab reduced time to onset of 12-week confirmed disability progression risk by 24% (*p* = 0.0321) compared with placebo. Timed 25-foot walk improved after 120 weeks (*p* = 0.04).

### Biotin

Vitamin B7-biotin activates acetylCoA carboxylase, a potentially rate-limiting enzyme in myelin synthesis and subsequently may help inducing remyelination (80). Indeed, a pilot study with 23 SPMS and PPMS patients reported improvement in clinical, radiological, or electrophysiological parameters in 91.3% of the patients receiving high dose (100–300 mg) biotin (80). Very recently, the results of the phase 3 MS-SPI study with its extension phase were published revealing that the primary end point “reversal of disability” was met in 13.2% of the study population (progressive MS) at month 12. Secondary end points like slowing of the EDSS progression were also met with acceptable tolerability and side effects profile (3) making biotin the first remyelinating agent to possibly enter the market.

### Laquinimod

Laquinimod is an orally available carboxamide derivative with multimodal mechanism of action rendering it both anti-inflammatory and neuroprotective (81); laquinimod reduces inflammatory cells in the brain (Th1 and Th17), shifts the cytokines into anti-inflammatory profile, promotes monocytes/macrophage maturation into regulatory subtypes, and modulates the dendritic cells reducing their ability to induce the CD4+ cells (82). The unexpected discrepancy between the modest effect on relapse rates and the unprecedented reduction of disease progression in clinical studies suggests a novel neuroprotective effect of laquinimod. Indeed, laquinimod increases brain-derived neuroprotective factor (83) and inhibit the inflammatory response of astrocytes and microglia leading to reduction of the axonal damage (84). Currently, a phase 2 clinical trial in PPMS (ARPEGGIO, NCT02284568) is recruiting patients, and the results are expected in September 2017.

### Simvastatin

In the MS-STAT study, 80 mg/day simvastatin was reported to cut the annualized brain atrophy rates in patients with SPMS by almost the half in a placebo-controlled randomized clinical trial (85). Simvastatin exhibits its immunomodulatory action by impacting the Th1 and Th17 as well as by modulation the dendritic cells (86, 87).

### EBV-Directed Therapies

Adoptive immunotherapy with autologous T cells expanded *in vitro* with AdE1-LMPpoly increased survival in patients with the EBV-associated carcinoma (88). Pender et al. applied the same approach in one SPMS patient with EDSS score of 8.0 leading to clinical and radiological improvement without serious side effects (89), a novel approach targets a mechanism provoking the autoimmune response in MS itself not the immune system generally (23). Furthermore, vaccination of seronegative individuals with recombinant gp350 may be considered as a novel “primary prophylaxis” to reduce the incidence of MS (23).

## Better Understanding, Better Treatment

Despite our increasing knowledge and better understanding of the underlying mechanisms, many questions remain open. Accumulating evidences support considering PPMS as a part of the MS spectrum. However, there is no solid explanation of what exactly drives the development of the clinical phenotypes. In our opinion, the core of MS may be a slowly progressive low-degree inflammatory process driven by autoreactive apoptosis-resistant EBV-infected B cells that manifests itself clinically in genetically predisposed individuals only after a specific age threshold is exceeded. In presence of other factors (like vitamin D deficiency), a superimposed fluctuating high-grade inflammatory process appears in younger age and manifests itself in the form of recurrent exacerbations. Evidences supporting this hypothesis are (1) more than half of MS patients suffer from PMS (either as PPMS from the beginning or SPMS), (2) the striking clinical and pathological similarities between PP and SPMS, (3) the almost universal positive EBV status in MS patients, (4) the presence of EBV-infected B cells in brain and meninges of MS patients, perivascular spaces, and parenchyma, (5) the well-known change in age-dependent host response to latent EBV infection, (6) the success of B cell-depleting agents in RRMS and PPMS, (7) the “preliminary” success of T-cell-based therapy against EBV-infected B cells in SPMS, (8) the presence of vitamin D deficiency in RRMS but not PPMS patients and its well-described effect on the relapse rate but not disease progression, and finally, (9) almost all pathological aspects of the progressive phase like MiA, iron accumulation, mitochondrial dysfunction, involvement of the NAWM and NAGM, cortical and cerebral atrophy, as well as meningeal infiltration can be detected very early in the disease course even in CIS patients (55, 90–93). Further work is needed to prove the exact role of EBV in PMS forms, to characterize the B_Auto_ population and how do they differentiate, and at last to explain the role of different risk factors in PMS and their interactions in different populations.

**Figure 3:**
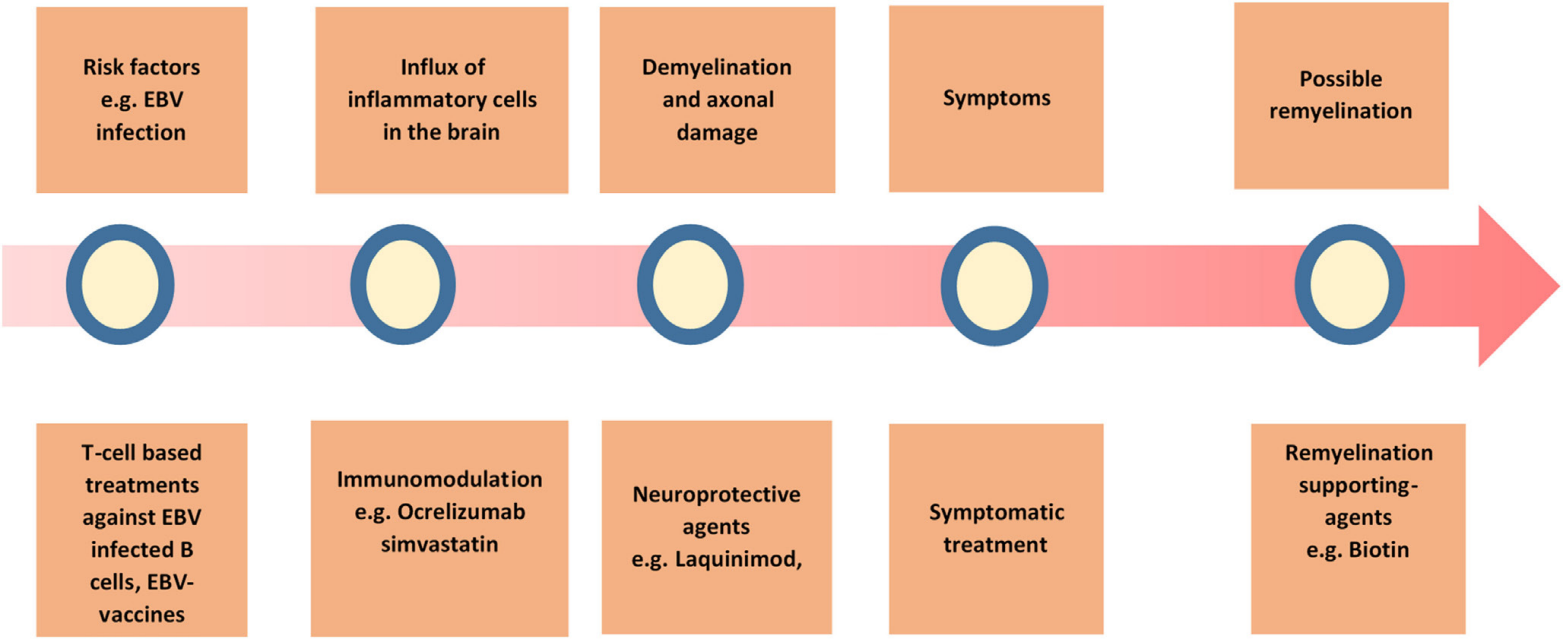
**Overview of the possible treatment strategies in primary progressive multiple sclerosis (PPMS)**. A summary of the current and possible treatment strategies in PPMS.

The current therapy options for PPMS are promisingly increasing with upcoming possibilities of targeting different aspects of the disease (Figure 3). Combination of different treatments may be a viable approach in the future, considering the suboptimal effect of every single treatment alone so far.

## Summary

Primary progressive multiple sclerosis is considered a relatively rare, but very challenging phenotype in the care of MS patients. Our current knowledge supports an underlying inflammatory-driven neurodegenerative process. Autoreactive EBV-infected B cells are essential to drive the progressive inflammation. The results of ocrelizumab and biotin announce the beginning of a new era with more therapies are eventually coming to the market in next years.

## Author Contributions

AA formulated the main concept, reviewed the published data, postulated the mentioned hypothesis, and drafted the manuscript. HT supervised and reviewed the article. MW reviewed the article.

## Conflict of Interest Statement

The authors declare that the research was conducted in the absence of any commercial or financial relationships that could be construed as a potential conflict of interest. The handling editor declared a past co-authorship with one of the authors, HT, and states that the process nevertheless met the standards of a fair and objective review.
